# Extracting structural information of Au colloids at ultra-dilute concentrations: identification of growth during nanoparticle immobilization†

**DOI:** 10.1039/c9na00159j

**Published:** 2019-05-21

**Authors:** George F. Tierney, Donato Decarolis, Norli Abdullah, Scott M. Rogers, Shusaku Hayama, Martha Briceno de Gutierrez, Alberto Villa, C. Richard A. Catlow, Paul Collier, Nikolaos Dimitratos, Peter P. Wells

**Affiliations:** School of Chemistry, University of Southampton Highfield Southampton SO17 1BJ UK P.P.Wells@soton.ac.uk; UK Catalysis Hub, Research Complex at Harwell, Rutherford Appleton Laboratory Harwell Didcot OX11 0FA UK; Department of Chemistry, Center for Foundation Science, National Defense University of Malaysia Sungai Besi Camp 57000 Kuala Lumpur Malaysia; Department of Chemistry, University College London 20 Gordon Street London WC1H 0AJ UK; Diamond Light Source, Harwell Science and Innovation Campus Chilton Didcot OX11 0DE UK; Johnson Matthey Technology Centre Sonning Common Reading RG4 9NH UK; Dipartimento di Chimica, Universitá degli Studi di Milano via Golgi 19 20133 Milano Italy; Cardiff Catalysis Institute, School of Chemistry, Cardiff University Cardiff CF10 3AT UK; Dipartimento di Chimica Industriale “Toso Montanari”, Alma Mater Studiorum Università di Bologna Viale Risorgimento 4 40136 Bologna Italy nikolaos.dimitratos@unibo.it

## Abstract

Sol-immobilization is increasingly used to achieve supported metal nanoparticles (NPs) with controllable size and shape; it affords a high degree of control of the metal particle size and yields a narrow particle size distribution. Using state-of-the-art beamlines, we demonstrate how X-ray absorption fine structure (XAFS) techniques are now able to provide accurate structural information on nano-sized colloidal Au solutions at μM concentrations. This study demonstrates: (i) the size of Au colloids can be accurately tuned by adjusting the temperature of reduction, (ii) Au concentration, from 50 μM to 1000 μM, has little influence on the average size of colloidal Au NPs in solution and (iii) the immobilization step is responsible for significant growth in Au particle size, which is further exacerbated at increased Au concentrations. The work presented demonstrates that an increased understanding of the primary steps in sol-immobilization allows improved optimization of materials for catalytic applications.

## Abstract 2

This paper describes the structural characterization of ultra-dilute colloidal Au nanoparticle solutions using X-ray absorption spectroscopy (XAS) and the particle growth during immobilization.

## Graphical abstract

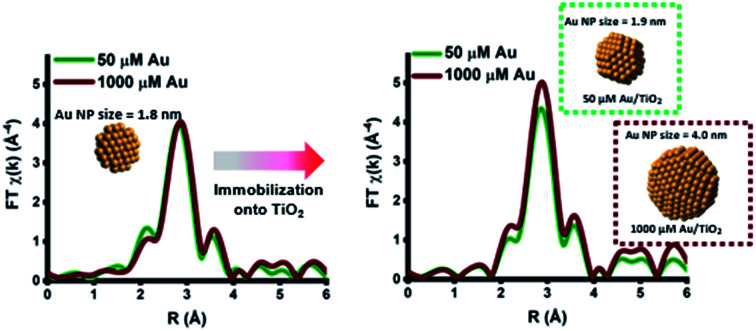

## Introduction

The study of supported metal nanoparticles (NPs) is a cornerstone of heterogeneous catalysis; their reduced size, distribution of surface sites, and interaction with metal oxide supports afford enhanced catalytic properties as well as providing effective use of noble metals.^1–5^ Within the various routes typically used to produce supported metal NPs, the application of sol-immobilization is becoming increasingly popular; it offers a high degree of tunability through the nature of stabilising or reducing agents,^6–11^ temperature of synthesis,^5,12–16^ solvent system,^5,16,17^ or concentration of the metal precursor salt.^18^

There have been many elegant studies that have assessed the influence of these parameters, primarily relying upon advanced electron microscopy to provide structural information on the final supported catalyst.^3,19,20^ However, to understand better the preparation of supported NPs through sol-immobilization, further insights into all steps in the process are needed. Au NPs are one of the most intensely studied nano-particulate systems due to the wide range of potential applications, from medicine to catalysis.^21^ Recent approaches to study the structural properties of colloidal solutions of Au NPs, have utilised high brilliance X-rays, produced by synchrotron radiation, for small angle X-ray scattering (SAXS) and X-ray absorption fine structure (XAFS) investigations. Much work in this area has followed the formation of Au NPs using time-resolved studies;^21–26^ the intention is to understand the principal steps in the evolution from defined precursors to NP entities. Using XAFS, these studies have assessed the formation of Au NPs from precursor Au solutions with concentrations ranging between 7 and 100 mM,^21,25–31^ with data acquisition times ranging between minutes and hundreds of ms. They have continually increased their sophistication, moving from simple sample environments, such as a large volume cells, towards precision engineered microfluidic reactors^28,29^ or acoustic levitation systems.^21^

Other than XAFS, SAXS has proved successful in studying colloidal Au NP systems.^21,24^ It allows for very fast measurements, in the order of milliseconds, and is able to provide information regarding the particle size and shape during the reaction procedure.^24,32^ However, the resolution for SAXS is limited to sizes ≥ 1 nm,^33^ below which meaningful data cannot be extracted. Moreover, a sufficient concentration, ≥200 μM,^23^ is needed to achieve the required electronic contrast. As a consequence, the concentrations of Au precursors employed in these XAFS and SAXS studies do not always reflect the typical conditions found in the recent literature for the preparation of Au NPs, *e.g.* 5–100 μM.^16,34–41^ At such low concentrations, the application of these forms of characterization becomes challenging.

Furthermore, these studies do not explore the changes to the properties of the NPs once they have been supported. It is well known that there is a special interplay between metal NPs and their supports; the strong metal-support interaction,^42^ and wettability,^43^ influence the properties of the NPs once immobilised.^43–46^ To understand how best to optimise the catalytic performance of supported NPs it is important to separate the contributions of synthesis conditions during the colloidal step and immobilization on the resultant colloidal NP structures.

**Fig. 1:**
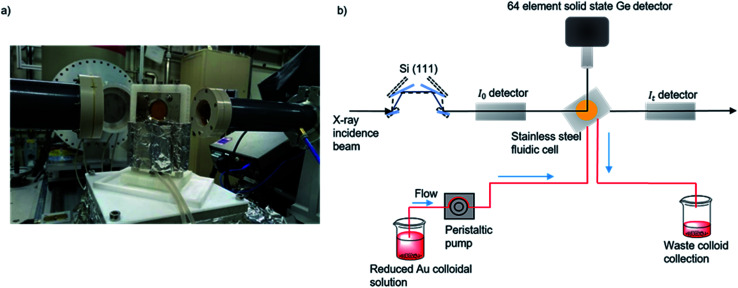
(a) Photograph of the *in situ* cell on the I20 beamline. (b) Experimental schematic for data acquisition of colloidal Au XAFS at the I20 beamline at the Diamond Light Source, Didcot, UK. A continuous flow of preformed colloid was pumped through PTFE tubing and XAFS data was acquired in fluorescence mode by a solid state Ge detector.^48,66^

In this work, XAFS studies (Fig. 1) of colloidal Au solutions prepared at different temperatures (1 °C, 25 °C, 50 °C, 75 °C) and concentrations (50 μM, 100 μM, 1000 μM) have been performed. This study assesses the structural properties of colloidal Au solutions at more relevant concentrations than previously reported and allows for the unique contributions of colloidal Au generation and the subsequent immobilization step to be disentangled.

## Experimental section

### Materials and methods

#### Catalyst preparation

Preparation of colloidal and supported Au NPs was performed using a conventional sol-immobilization method; concentration of the Au metal salt precursor and the temperature during the reduction processes were altered. HAuCl_4_ was used in preparation of the colloidal solutions at specific concentrations using deionised water (18.2 MΩ cm) (50 μM, 100 μM and 1000 μM); PVA was added as the stabilising agent (1 wt% solution, Aldrich, MW = 9000–10 000 g mol^−1^, 80% hydrolysed, PVA/Au (wt. ratio = 0.65)). Solutions of NaBH_4_ (0.1 M) >96%, Aldrich, NaBH_4_/Au (molar ratio = 5) were subsequently added drop-wise over the course of a minute, under continuous stirring evolution of dark red sols was observed. Formation of colloidal Au NPs occurred after a 30 minute time period and studied by UV-vis spectroscopy and XAFS. TiO_2_ stabilised colloids (0.99 g P25, Degussa), were prepared, with a calculated amount of TiO_2_ added to give a final metal loading of 1 wt%. The supported Au/TiO_2_ mixture was acidified to pH 1–2 using H_2_SO_4_ before a 60 minute period under vigorous stirring, ensuring full immobilization of the Au NPs on TiO_2_; the mixture was filtered, washed with distilled water and dried overnight at room temperature. The different temperature and concentrations used in synthesis of the catalysts in this paper are listed in Table 1.

**Table 1:** Au SPR band maximum and average NP diameters calculated through TEM and EXAFS analysis

Sample name	Temperature of preparation (°C)	[Au] (μM)	UV-vis max (nm)	Colloidal Au NPs			Au/TiO_2_		Av. EXAFS NP size^a^ (nm (ref. 59))
				Av. TEM NP size (nm)	CN_Au–Au_	Av. EXAFS NP size^a^ (nm (ref. 59))	Av. TEM NP size (nm)	CN_Au–Au_	
A_1_	1	100	492	3.0 ± 0.9	9.3 ± 0.6	1.6	2.7 ± 0.7	10 ± 0.7	2.0
B	1	50	495	—	9.2 ± 0.9	1.5	2.3 ± 0.6	9.5 ± 0.8	1.6
C	1	1000	498	4.6 ± 1.5	9.4 ± 0.4	1.6	4.0 ± 1.0	10.3 ± 0.5	2.3
A_2_ (ref. 16)	25	100	493	—	9.8 ± 0.5	1.9	2.9 ± 0.9	8.4 ± 0.3	1.4
A_3_ (ref. 16)	50	100	500–510	—	10.2 ± 0.7	2.2	2.8 ± 0.9	9.5 ± 0.3	1.9
A_4_ (ref. 16)	75	100	538	—	10.3 ± 0.7	2.3	3.3 ± 0.8	11.2 ± 0.4	—

a The error in the process of calculating particle size from 1^st^ shell coordination number has an intrinsic error of 0.1 nm. CN_Au–Au_ refers to the Au–Au coordination number found by fitting the experimental EXAFS data using the *Artemis* software package,^49^ NP EXAFS fits are shown in Fig. S6. The remaining Au first shell fitting parameters for all samples can be found in Table S1.

#### UV-visible spectroscopy

Evolution of the colloidal hydrosols were analysed by UV-vis spectroscopy to determine the position of the gold surface plasmon resonance (SPR) band. Generation of the Au colloid SPR bands were recorded using a Shimadzu UV-1800 spectrometer in a quartz cuvette after 30 min of colloid formation.

#### X-ray absorption fine structure (XAFS)

XAFS measurements of both colloidal and TiO_2_ supported Au NPs at the Au L_3_-edge were performed on the scanning branch of the I20 beamline at the Diamond Light Source, Didcot, U.K. Colloidal Au NPs were studied in a continuous flow using an adapted stainless steel cell (Fig. 1); 1/16^th^ outer diameter PTFE tubing and peristaltic pump were used to continuously move the solution through the cell, with Kapton window in place as X-ray transparent windows. A low flow rate of 1 mL min^−1^ was applied to limit beam damage to the Au NPs^47^ and also prevent the evolution of H_2_ gas from excess NaBH_4_ reaction along the tubing walls. 1 wt% Au/TiO_2_ powder catalysts were studied *ex situ* and prepared for testing as 8 mm pellets and placed in a X-ray transparent cell. *In situ* XAFS measurements were made in fluorescence mode using a Si(111) four-bounce monochromator, data was collected through a 64 element Ge fluorescence detector with the xpress2 digital pulse processor.^48^ Scans were performed at a time resolution of 43 min per spectrum (*K*_max_ = 18), with 6 scans collected for the colloidal solutions and 3 for the powder catalysts. Processing and normalization of the absorption spectra and analysis of the extended X-ray absorption fine-structure (EXAFS) were accomplished using IFEFFIT with the Horae package (Athena and Artemis).^49,50^ Experimental determination of the amplitude reduction factor, *S*_0_^2^, was achieved through EXAFS data analysis of the Au foil spectrum, giving the fixed parameter of 0.83. EXAFS data fittings were made with *R*-space within a fit range of 1.74 < *R* < 3.37 Å (for both colloidal and TiO_2_ supported Au NPs) and a *k*-space of 3 < *k* < 11.5 (colloidal Au NPs) and 3 < *k* < 14 (TiO_2_ supported Au NPs).

#### Transmission electron microscopy (TEM)

Preparation of the samples for TEM imaging required the initial dispersion of the catalyst in high purity ethanol utilizing ultra-sonication over a 30 minute period, 40 μL of this solution was dropped and evaporated onto a holey carbon film supported by a 300 mesh Cu TEM grid. A JEOL JEM 2100 EM was used to study the Au/TiO_2_ samples.

#### Scanning TEM high angle annular dark field (STEM HAADF)

For imaging of the colloidal Au NPs, a drop of the colloid solution was placed on a holey carbon TEM grid and allowed to dry in air. Samples with Au concentrations 50, 100 and 1000 μM were examined using a JEM 2800 (Scanning) TEM. High angle annular dark-field (HAADF) imaging in scanning mode used an off-axis annular detector. Secondary electron signals were acquired simultaneously with other STEM images, providing topological information of the samples. X-ray emission detection was performed of the sample in scanning mode.

## Results and discussion

Ultraviolet-visible (UV-vis) spectroscopy has been used to study both the Au precursor solution (reduction rate) and the formation of Au NPs through the formation of surface plasmon resonance (SPR) band before immobilization.^51–53^ The position of the SPR peak, shown in Fig. S1,†*λ*_max_, is related to the particle size and shape of NPs; a shift to higher wavelength is indicative of an increase in Au particle size.^51^ The intensity of the band can also give a measure of particle size, as larger particles give more intense peaks, however, in this study there is a correlation of parameters with both particle size and concentration contributing towards this. It has been previously reported that the size of Au NPs supported on TiO_2_ can be tailored by systematic control of the temperature of reduction of Au precursor salts; an increase in reduction temperature has the effect of increasing the particle size of the supported Au NPs.^51–53^ This observation was repeated in this work (Table 1); as the temperature increases from 25 °C to 75 °C (A_1_–A_4_), the position of *λ*_max_ shifts from 493 nm to 538 nm, indicating a growth in particle size. The first series of Au colloidal solutions were all prepared using an initial Au concentration of 100 μM. However, for the 1 °C reduction temperature two additional concentrations, 50 μM and 1000 μM (B and C), were also investigated. For this variable concentration series, there were no discernible changes to the SPR *λ*_max_, the broad nature of these bands makes it difficult to extract any quantifiable assessment of the particle size characteristics.^54^

**Fig. 2:**
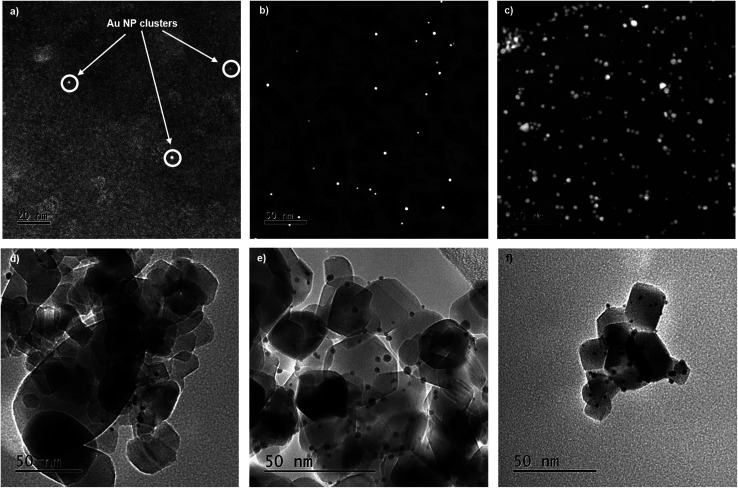
(a–c) STEM HAADF of Au colloids prepared using increasing Au concentration at 1 °C, (a) 50 μM (B) 100 μM (C) 1000 μM droppered onto a holey carbon TEM grid, (d–f) TEM images of the TiO_2_ supported colloidal Au, with immobilization performed at 1 °C (d) 50 μM (e) 100 μM (f) 1000 μM.

When analysing the properties of colloidal NPs at concentrations comparable to those employed previously,^55^ UV-vis and TEM both provide an incomplete description. The broad nature of the Au SPR band and the challenge of measuring colloidal solutions using TEM without causing aggregation of the colloidal NPs (Fig. 2a–c), does not capture sufficient detail to make a reliable comparison between supported and unsupported Au NPs. Recent advances through the increased flux of modern insertion device-based beamlines and multi-element fluorescence detectors, have allowed XAFS to provide a suitable tool for studying both unsupported and supported NP catalysts while avoiding beam damage (Fig. 2, S7 and S8†).

**Fig. 3:**
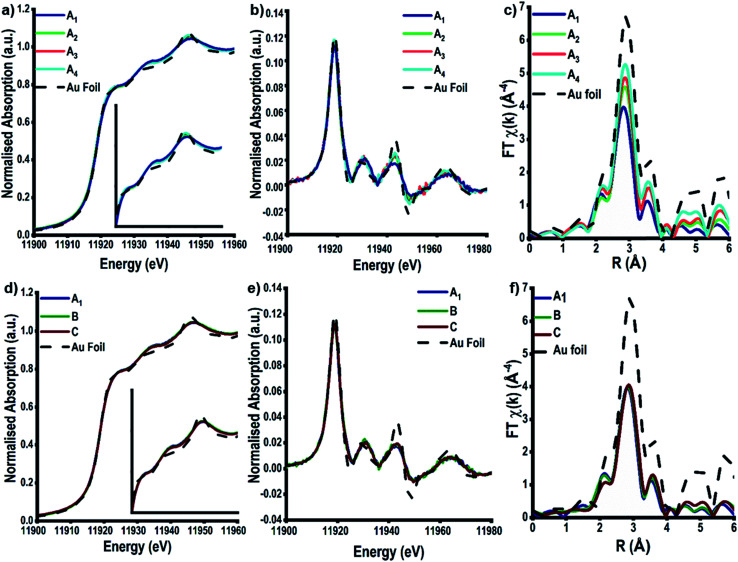
XAFS taken at the Au L_3_-edge of the colloidal Au samples; (a & d) XANES spectra for the colloidal Au detailing change as a result of increasing synthesis temperature and Au concentration, (b & e) the normalised first derivative of the absorption for the temperature and Au concentration influenced colloids respectively and (c & f) experimental Fourier transform (FT) chi(*k*) data of the corresponding EXAFS signals for colloidal Au showing the influence of temperature and Au concentration.

The high energy and stability of the I20-scanning beamline at Diamond Light Source, U.K., has been used to acquire reliable XAFS data, at the Au L_3_-edge, of our colloidal and TiO_2_ supported Au NPs (Fig. 3).^48^ The normalised XANES spectra (Fig. 3a) of colloidal Au prepared at variable temperature (samples A_1–4_) are characterised by the absence of an intense white line peak at ∼11 915 eV, which is indicative of Au^0^ being the only species in solution; the white line arises due to dipole allowed 2p to 5d transitions, as Au^0^ has a full d-shell there is not an intense main edge transition, as seen in Fig. S2.†^29,31,56^ On assessing the colloidal solutions as a function of temperature, we observe that the feature at ∼11 946 eV increases in intensity; the change in this feature is more pronounced and easier to observe when plotting the XANES data in its 1^st^ derivative form (Fig. 3b). This continuum feature has been ascribed previously to Au–Au multiple scattering interactions and is linked to the particle size of the Au NPs.^29,57,58^ The larger the particle size, the higher the amount of multiple scattering from Au–Au interactions, resulting in an increase in the intensity of this feature. Varying the concentration of Au precursors (A_1_–C), however, does not appear to have an effect on the normalised XANES spectra (Fig. 3d), or the first derivative spectra (Fig. 3e). The absence of the multiple scattering post edge feature suggests that for all three samples, A_1_–C, the particle size is ≤2 nm.^29,57,58^

The EXAFS analysis, however, can be used to obtain quantitative information on the colloidal NP size. Fig. 3c shows the *k*^3^-weighted forward Fourier transform EXAFS spectra for samples A_1_–A_4_, EXAFS chi data can be found in Fig. S4.† It is evident that there is a change in intensity of the feature located at ∼2.8 Å as the temperature of reduction increases. This feature arises as a consequence of 1^st^ shell Au–Au scattering interactions and the intensity of this peak can be correlated to an increase in the Au–Au coordination number (CN), which, in turn, shows the trend in particle size. With smaller Au NPs having a higher surface fraction there is a greater proportion of under coordinated surface Au atoms, leading to decreases in the average Au–Au CN.^60^

Calculations of the average Au NP sizes, were performed using methods reported by Beale *et al.*^59^ XAFS spectra were modelled based on different particle sizes assuming all Au NPs are; (1) spherical in shape, (2) FCC in packing and (3) <3 nm in diameter. A similar approach was used for samples A_1_, B, and C, where no changes can be seen in the Fourier transform EXAFS spectra, or in the generated fitting parameters.

The particle sizes obtained from the fits confirmed that, within the error of the measurement, adjusting the concentration of the Au, between 50–1000 μM, precursor does not alter the size of the colloidal NPs produced. This is an important observation as it is already known that for sol-immobilization, increasing the concentration of initial precursor solutions manifests itself as an increase of supported Au NP sizes.^55^ However, we can now confirm that this effect occurs only during the immobilization phase and not in the preformed colloidal NPs.

To assess the effect of immobilization, the corresponding Au/TiO_2_ materials (from solutions of A_1_, B, & C) were prepared and XAFS data were acquired (Fig. S6†); as the Au precursor concentration increases from 50–1000 μM, the NPs size increases from 1.6 to 2.3 nm. This in direct contrast with the results obtained for the colloidal Au NPs, where all the particles have approximately the same particle size.

A possible explanation for this behaviour could be attributed to the mobility of NPs on the support surface. As the synthesis temperature reaches the Hüttig (TH = 0.3*T*_melting_ [*K*]) and Tamman temperatures (TT = 0.5*T*_melting_ [*K*]), the surface atoms and bulk atoms respectively become mobile,^61^ constituting a mechanism for particle mobility.^62^ For NPs ∼2 nm in size, as observed in the colloidal solutions, the *T*_melting_ can be as low as ∼330 °C,^63^ giving TH ∼ −93 °C and TT ∼ 25 °C.^64^ We suggest that with a higher concentration of the Au precursor and subsequent increase in surface NP density, coupled with slow migration of the mobile surface atoms yields NP growth through Ostwald ripening.^65^ Contrary to this, when preparing the colloid with a lower concentration, NPs are dispersed with larger interparticle distances, revealing negligible changes to NP size pre- and post-immobilization.

A comprehensive explanation for this behaviour has yet to be given in literature, but a further plausible reason could be a saturation of anchoring sites on the surface of the support, which, in turn, force the coalescence of the non-anchored nanoparticles. In the case presented the general Au wt% does not change amongst the samples, however, a possible change in local particle density during the immobilization phase could cause a growth in the particles due to a similar phenomenon, as the one shown by changing the metal loading.^34^ Regardless of the cause for the increase in size, it is clear that the in order to fully design and optimise supported NPs it is crucial to understand the relatively underexplored immobilization process; this is a key point in directing the resultant NP properties.

## Conclusions

This work demonstrates that XAFS is a suitable method to extract quantifiable structural data on ultra-dilute solutions of colloidal Au NPs (50 μM), akin to those commonly used in sol-immobilization processes. These studies have revealed several key findings: (i) the particle size of the colloidal Au is dependent on the temperature of reduction, with lower temperatures generating smaller particles, (ii) the concentration of Au precursor does not influence the size of the formed colloidal NPs within the range of 50 to 1000 μM and (iii) the immobilization stage of synthesis influences the Au particle size growth processes. Therefore, controlling and optimising the immobilization step is of paramount importance for the usage of low/high diluted metal colloidal solutions to synthesize size/shape supported metal nanoparticles.

## Funding sources

All authors acknowledge UK Catalysis Hub Consortium and EPSRC (Grants EP/K014706/1, EP/K014668/1, EP/K014854/1, EP/K014714/1, and EP/I019693/1). D. D. and P.·P.·W. acknowledge further support through the STFC ST/R002754/1.

## Conflicts of interest

There are no conflicts to declare.

## Abbreviations

NP: Nanoparticle
μM: Micromolar (10^−6^ molar)
SPR: Surface plasmon resonance
CN: Coordination number

## Data access statement

All data supporting this study are openly available from the University of Southampton repository at https://doi.org/10.5258/SOTON/D0921.

## Supplementary Material

NA-001-C9NA00159J-s001
