# Supplementary material for: Extracting structural information of Au colloids at ultra-dilute concentrations: identification of growth during nanoparticle immobilization
Source: Nanoscale Adv. 2019 May 21;1(7):2546–52. doi: 10.1039/c9na00159j (PMC9419830; doi:10.1039/c9na00159j)
Supplement: NA-001-C9NA00159J-s001 [file NA-001-C9NA00159J-s001.pdf]

Electronic Supplementary Material (ESI) for Nanoscale Advances.
This journal is © The Royal Society of Chemistry 2019

## SUPPORTING INFORMATION

**Table S1.** Au $L_3$-edge EXAFS first shell fitting parameters of the Au colloids and 1 wt. % Au/$TiO_2$ catalysts prepared at different temperatures and Au concentrations.

| Sample | $E_0$ / eV | $\sigma^2$ / Å$^2$ | $R_{factor}$ | R / Å |
|---|---|---|---|---|
| Au foil | 4.3(2) | 0.008 | 0.0057(1) | 2.85(3) |
| Colloidal Au NPs | | | | |
| $A_1$ | 3.6(5) | 0.0108(6) | 0.0084(1) | 2.806(5) |
| $A_2$ | 4.3(4) | 0.0096(5) | 0.0061(1) | 2.833(4) |
| $A_3$ | 4.2 ± 0.5 | 0.0096(6) | 0.009(1) | 2.839(4) |
| $A_4$ | 4.4 ± 0.5 | 0.009(6) | 0.010(1) | 2.843(4) |
| B | 3.6 ± 0.6 | 0.010(9) | 0.018(2) | 2.812(6) |
| C | 4.7 ± 0.4 | 0.0109(6) | 0.00059(1) | 2.828(4) |
| $TiO_2$ supported Au NPs | | | | |
| $A_1$ | 4.1 ± 0.5 | 0.01024(7) | 0.026(4) | 2.836(4) |
| B | 4.2 ± 0.5 | 0.0104(8) | 0.0024 | 2.836(5) |
| C | 4.6 ± 0.3 | 0.0095(5) | 0.0008 | 2.848(3) |

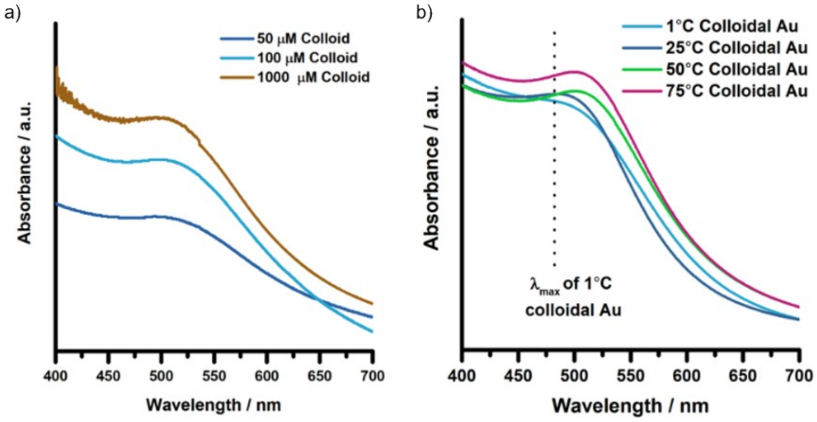


**Figure S1.** UV-Vis spectra showing the formed Au SPR bands after reduction of the $HAuCL_4$ precursor solutions prepared at: a) varied Au concentration and b) increasing temperature of reduction.

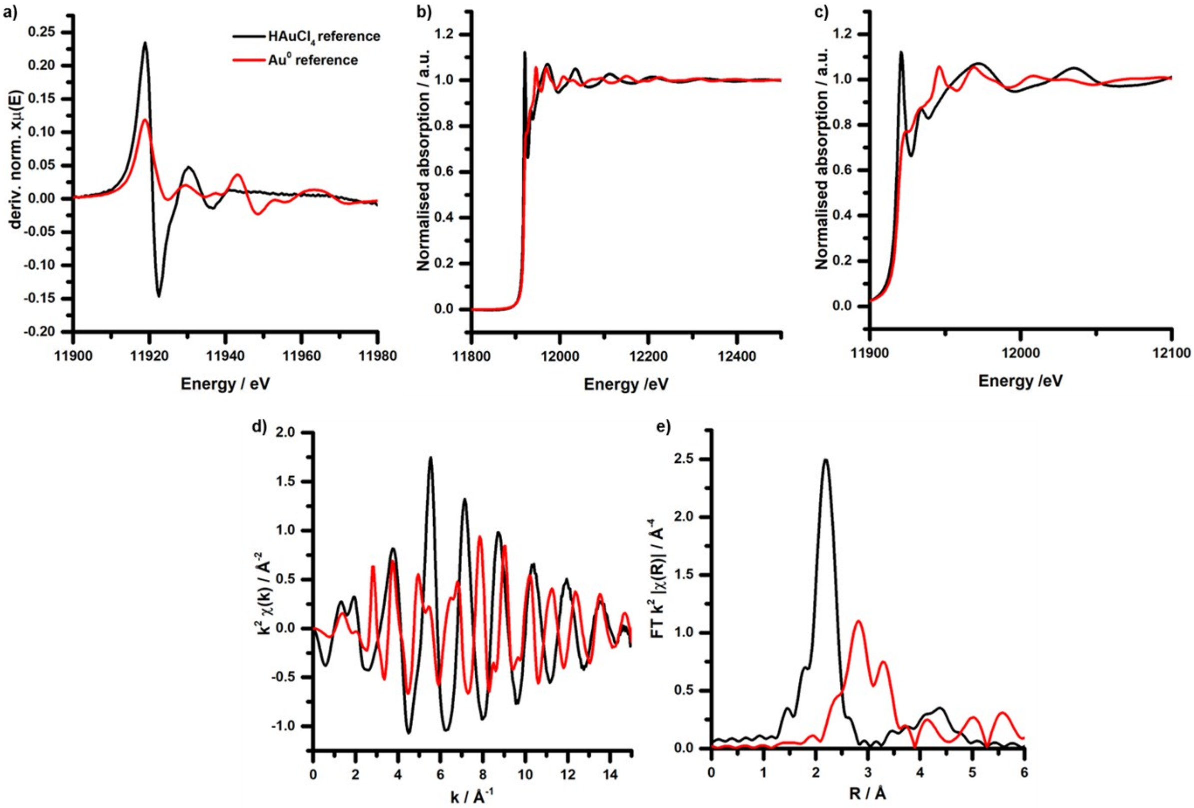


**Figure S2**. Acquired Au *L3*-edge XAFS data of an $Au^{3+}$ reference (supplied by G. Malta[1]), spectra was measured on the B18 beamline, Diamond Light Source, Didcot, U.K.[1] Also shown is the Au $L_3$-edge XAFS data of a $Au^0$ foil reference measured on I20 at the Diamond Light Source, Didcot, U.K. a) Shows comparison of the normalised first derivative of μ(E) for both the $Au^{3+}$ ($HAuCl_4$) and $Au^0$ species. b) Full comparable XAFS spectra of both Au references, c) XANES spectra of both Au species, with noticable depletion of the white line peak for $Au^0$ reference spectra. d) chi(k) space data for both $Au^{3+}$ and $Au^0$ reference compounds, e) shows the forward Fourier transform of the chi(k) data in S2D.

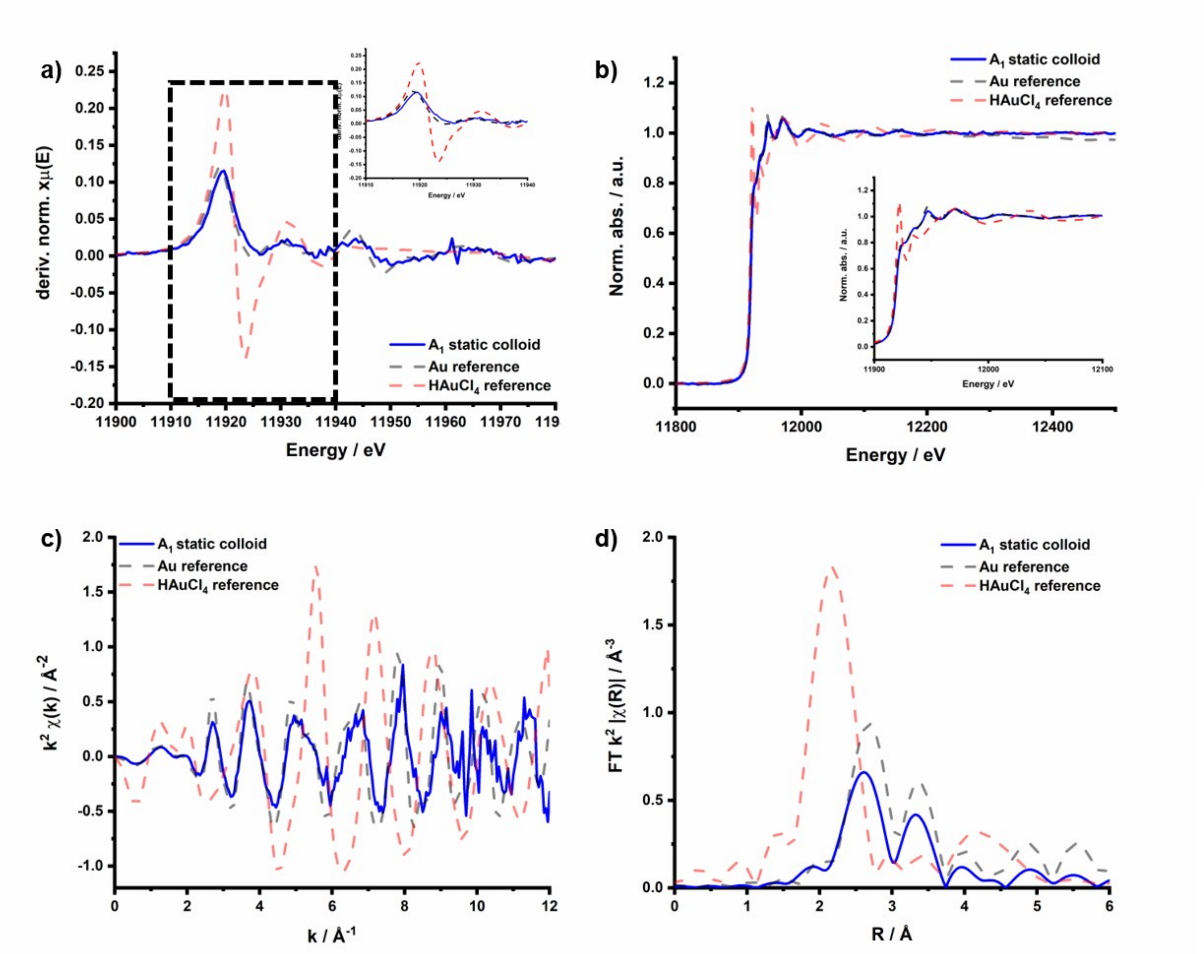


**Figure S3.** Acquired Au $L_3$-edge XAFS data of colloid $A_1$ (1°C and 100 μM [Au]) collected after leaving the colloid in the X-ray source beam path for a full scan (~ 48 minutes). The static colloid data is presented with references of the precursor salt ($HAuCl_4$) and zero-valent Au, all data was acquired at the Diamond Light Source (DLS) , Didcot, U.K. Both $A_1$ static colloid and Au reference datasets were acquired on the I20 scanning beamline of the DLS during the course of the experiment, and the $HAuCl_4$ reference was supplied by G. Malta and acquired on the B18 beamline at the DLS.[1] a) Comparison of the normalised first derivative of μ(E) for the references and static colloid, b) XANES spectra of Au references and static colloid, c) *k*-space data of $A_1$ compared to both Au reference materials, d) shows the Fourier transform of the *k* data in S3c, detailing the nearest neighbouring atomic species to the absorbing atom.

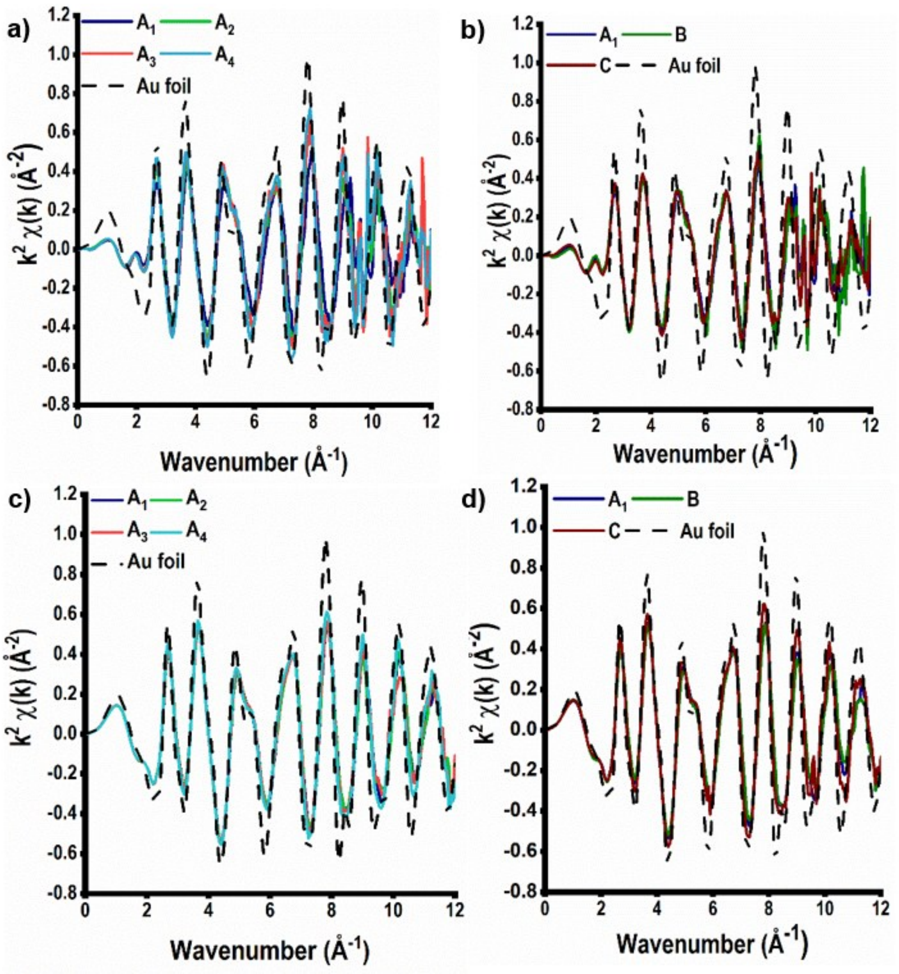


**Figure S4.** EXAFS chi data taken at the Au $L_3$-edge of both Au NP series; (a&b) colloidal Au NPs, (c&d) $TiO_2$ supported Au NPs.

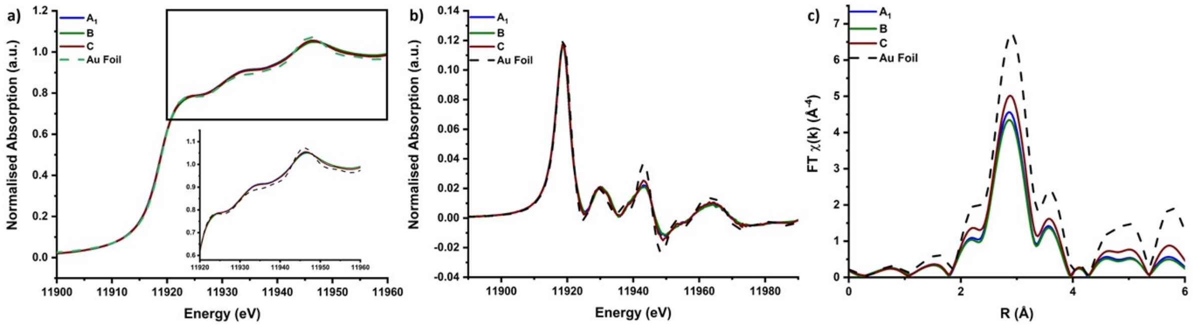


**Figure S5.** XAFS taken at the Au *L3*-edge of the TiO2 supported Au samples; a) XANES spectra for the supported Au NPs detailing change as a result of increasing Au concentration. b) The normalised first derivative of the absorption for Au concentration influenced supported NPs respectively, c) Experimental Fourier transform (FT) chi(k) data of the corresponding EXAFS signals for supported Au NPs showing the influence of Au concentration.

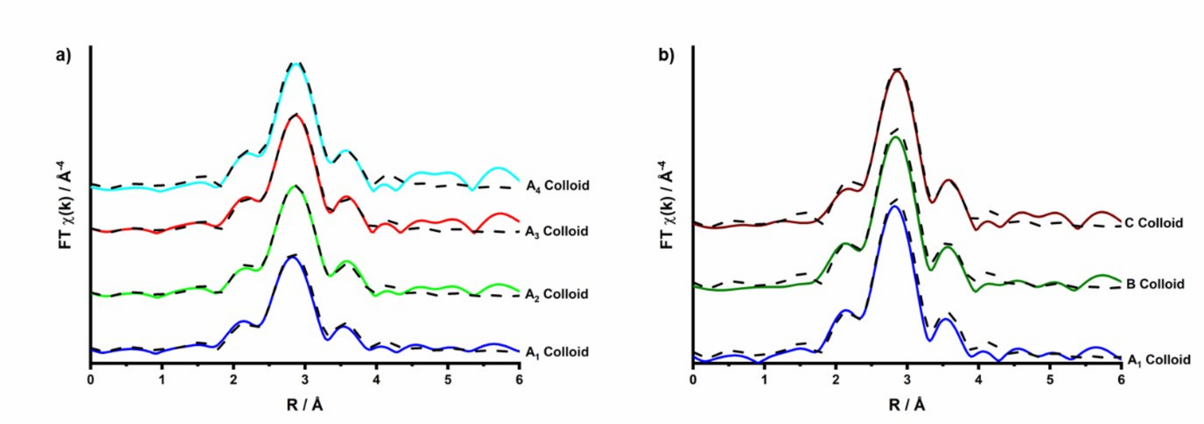


**Figure S6**. Fitted experimental Fourier transform k data of the corresponding EXAFS signals for colloidal Au NPs, synthesised using: a) an increase in the temperature of reduction and b) an increase in the concentration of Au present in the precursor solution.

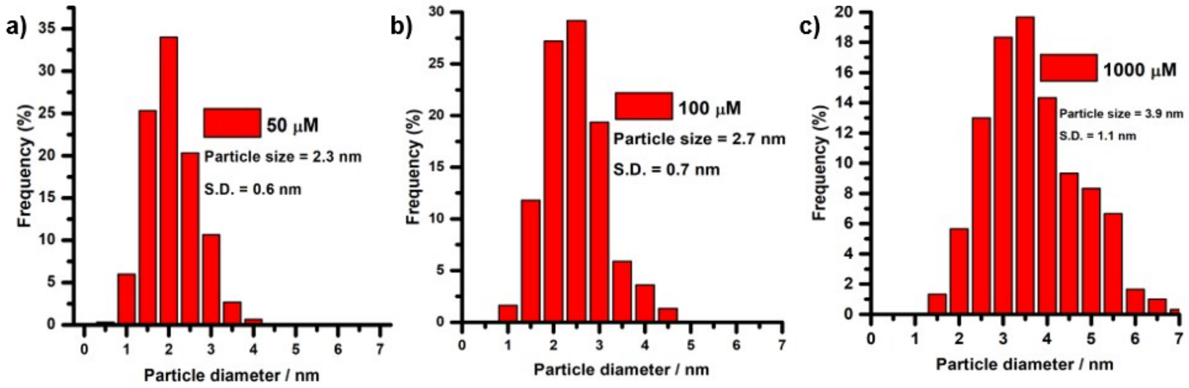


**Figure S7.** Histograms showing the average Au NP sizes and standard deviations calculated from TEM images of 1 wt. %Au/$TiO_2$ prepared with increasing Au concentration: a) 50, b) 100 and c) 1000 μM respectively.

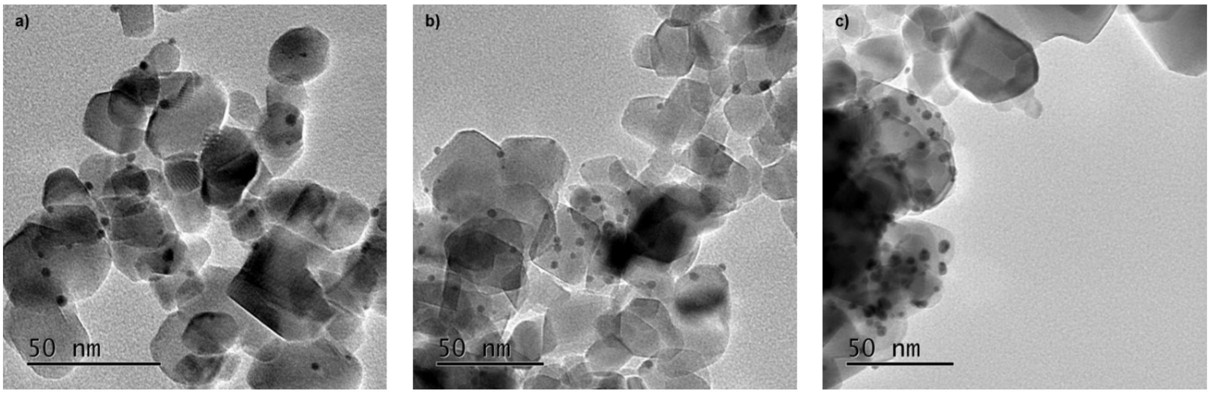


**Figure S8.** TEM images of 1 wt. %Au/$TiO_2$ prepared under increasing temperature of reduction: a) 25°C, b) 50°C and c) 75°C respectively.

1 G. Malta, S. A. Kondrat, S. J. Freakley, C. J. Davies, L. Lu, S. Dawson, A. Thetford, E. K. Gibson, D. J. Morgan, W. Jones, P. P. Wells, P. Johnston, C. R. A. Catlow, C. J. Kiely and G. J. Hutchings, *Science (80-. ).*, 2017, **355**, 1399–1403.
